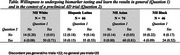# Attitudes toward biomarker disclosure in the setting of preclinical AD trials among racial and ethnic groups

**DOI:** 10.1002/alz70858_103566

**Published:** 2025-12-24

**Authors:** Christina M Magana‐Ramirez, Megan G Witbracht, Crystal M Glover, Kristin Harkins, Jason Karlawish, Daniel L Gillen, Josh D Grill

**Affiliations:** ^1^ University of California, Irvine, Irvine, CA, USA; ^2^ Institute for Memory Impairments and Neurological Disorders, University of California, Irvine, Irvine, CA, USA; ^3^ The UC Irvine Institute for Memory Impairments and Neurological Disorders, Irvine, CA, USA; ^4^ University of Pennsylvania Perelman School of Medicine, Philadelphia, PA, USA; ^5^ University of Pennsylvania, Philadelphia, PA, USA; ^6^ The UC Irvine Institute for Alzheimer's Disease Research Center, Irvine, CA, USA

## Abstract

**Background:**

Diverse communities remain underrepresented in Alzheimer's disease (AD) research, particularly clinical trials. Preclinical AD trials uniquely require cognitively unimpaired individuals to undergo and learn results of biomarker testing.

**Method:**

We studied racial and ethnic differences in willingness to undergo amyloid PET scans and learn results, both generally and for preclinical Alzheimer's disease trials. Participants (*n* = 257) were grouped as Hispanic/Latino (*n* = 66), Non‐Hispanic (NH) Asian (*n* = 74), NH Black (*n* = 46), and NH White (*n* = 71). Using logistic regression, we assessed willingness across contexts and examined discordant responses, controlling for confounding factors.

**Result:**

As seen in Table 1, the proportions of participants willing to undergo an amyloid PET and learn the results overall differed by group (Hispanic/Latino: 0.67, NH Asian: 0.86, NH Black: 0.70, NH White: 0.72; *p* = 0.034). Similarly, the proportions willing to undergo biomarker testing in the context of a preclinical AD trial differed by group (Hispanic/Latino: 0.68, NH Asian: 0.81, NH Black: 0.61, NH White: 0.79; *p* = 0.005).

When conditioning upon participants that indicated willingness to undergo biomarker testing outside of a clinical trial, we estimated that the odds of not being willing to undergo testing in the context of a trial was higher among the underrepresented groups as compared to the NH White group (Hispanic/Latino EST: 30.3863, 95 CI: [2.7909, 330.8355]; NH Asian EST: 2.9922, 95 CI: [0.2916, 30.7086]; NH Black EST: 28.4249, 95 CI: [3.0136, 268.1096]; *p* < 0.001 for a test of difference across all groups). Alternatively, conditional on being unwilling to undergo biomarker testing outside of a trial setting, we estimated that the odds of being willing to undergo testing in the context of a preclinical AD trial were observed to be lower among Hispanic/Latino and NH Black subpopulations when compared to the NH White subpopulation, though these difference were not statistically significant (Hispanic/Latino EST: 0.2276, 95 CI: [0.0159, 3.2580]; NH Black EST: 0.3446, 95 CI: [0.0431, 2.7535]; *p* = 0.495 for a test of differences across all groups).

**Conclusion:**

Our findings suggest race and ethnicity are associated with willingness to undergo biomarker testing and learn results, both generally and in preclinical Alzheimer's disease trials.